# Seroprevalence of *Toxoplasma gondii* and associated alterations in hematology and serum biochemistry of one-humped camels (*Camelus dromedarius*) in Pakistan

**DOI:** 10.14202/vetworld.2022.110-118

**Published:** 2022-01-23

**Authors:** Aamir Shehzad, Awais Masud, Tabassam Fatima, Fraz Munir Khan, Saifur Rehman, Mustofa Helmi Effendi, Lucia Tri Suwanti, Iahtasham Khan, Wiwiek Tyasningsih, Shah Faisal, Zain Ul Abadeen, Samreen Bibi

**Affiliations:** 1 Division of Microbiology, Faculty of Veterinary Medicine, University of Airlangga, Surabaya 60115, Indonesia; 2 Livestock and Dairy Development, Government of Punjab, Lahore, Pakistan; 3 Department of Microbiology, Faculty of Veterinary Sciences, University of Veterinary and Animal Sciences, Lahore, Pakistan; 4 Department of Parasitology, Riphah College of Veterinary Sciences, Riphah International University, Lahore, Pakistan; 5 Department of Veterinary Public Health, Faculty of Veterinary Medicine, University of Airlangga, Surabaya 60115, Indonesia; 6 Department Veterinary Parasitology, Faculty of Veterinary Medicine, University of Airlangga, Surabaya 60115, Indonesia; 7 Department of Clinical Sciences, Section of Epidemiology and Public Health, Jhang Campus, University of Veterinary and Animal Sciences, Lahore, Pakistan; 8 Department of Pharmacy Practice, Faculty of Pharmacy, University of Airlangga Surabaya 60115, Indonesia; 9 Department of Pathology, University of Agriculture Faisalabad, Pakistan; 10 Institute of Food Science and Nutrition, Faculty of Agriculture, University of Sargodha, Sargodha Division, Punjab, Pakistan.

**Keywords:** biochemistry, camel, hematology, public health, seroprevalence, *Toxoplasma gondii*

## Abstract

**Background and Aim:**

*Toxoplasma gondii* is an intracellular protozoan that infects humans and animals. This study aimed to estimate the seroprevalence of *T. gondii* and the associated alterations in hematology and serum biochemistry of one-humped camels (*Camelus dromedarius*) in Mianwali district, Pakistan.

**Materials and Methods:**

A total of 350 blood samples were obtained from male and female camels of different ages (≤3 years old, 4-6 years old, and ≥7 years old). To validate *T. gondii* antibodies, the collected samples were subjected to indirect enzyme-linked immunosorbent assay using purified recombinant micronemal protein 3 as an antibody catching antigen.

**Results:**

The prevalence of *T. gondii* was 50.2% higher in male camels than in female camels (16.5%) (p<0.001). Furthermore, the prevalence of *T. gondii* in camels was directly proportional to age (p<0.001). It was 63.33% (57/90) in camels of ≥7 years of age, 32.54% in 4-6 years old age group, and 23.08% in ≤3 years old age group. The hematological analysis of infected camels revealed a significant increase in the values of glucocorticoid-remediable aldosteronism, lymphocyte percentage, monocyte percentage (MONO%), corpuscular hemoglobin (MCH), and procalcitonin. Furthermore, substantially higher levels of liver enzymes alanine aminotransferase, aspartate aminotransferase, and the macro-mineral potassium were found in the serum of *T. gondii*-infected camels.

**Conclusion:**

The seropositivity of *T. gondii* is directly associated with the age and sex of camels, which may be considered as potential risk factors. Furthermore, *T. gondii* infection directly impacts the hemato-biochemistry of infected camels.

## Introduction

The one-humped camel (*Camelus dromedarius*) is found throughout Africa, South Asia, Australia, and the Middle East [1,2]. The global camel population is estimated to be approximately 35 million [3]. They are an important source of meat and milk in many African and Asian countries. Among camelids, the dromedary camels account for 95% of the world’s camel population and produce 2,852,213 tons of milk and 630,210 tons of meat per year [4-6]. They are one of the less well-studied animals in Pakistan. Pakistan has consid­erable importance among camel-raising nations, with an estimated population of 1.1 million camels [7]. The camel population is distributed throughout the country; the highest concentration is in Balochistan (41%), followed by Punjab (22%), Sindh (30%), and Khyber Pakhtoon Khwah (7%) [8]. In Punjab, Pakistan, there are two major camel breeds, Barela and Marecha, which can be found in the Thal desert region of Mianwali district [9]. Camels are known as the “ships of the desert;” they are an important mode of transportation in parts of the Thal desert, especially in the district of Mianwali. Camels can become infected with a variety of parasites, including approximately 10 protozoal infections, 48 helminth infections, and approximately 13 species of ectoparasite. The major protozoan genera involved in infection in camels include *Babesia, Balantidium, Besnoitia, Cryptosporidium, Eimeria, Neospora, Sarcocystis, Theileria, Trypanosoma*, and *Toxoplasma* [10]. *Toxoplasma gondii*, an apicomplexan parasite, causes toxoplasmosis in numerous mammals [11,12].

Camels acquire *T. gondii* infection by ingesting sporulated oocysts shed in the feces of cats and other wild animals [13]. Toxoplasmosis causes abortion [14]. The prevalence of *T. gondii* ranges from 3.12% to 90.9% in different areas of the world [15-17]. Serological tests have been proven to be a reliable method for detecting *T. gondii* infection in humans and animals [18,19]. Enzyme-linked immunosorbent assays (ELISAs) are well known for their sensitivity, flexibility, and cost-effectiveness [20,21]. Some recombinant proteins of *T. gondii* can be expressed in *Escherichia coli* by binding them to the specific antibodies of *T. gondii* and then can be used for the detection of antibodies of *T. gondii* during serodiagnostic studies [22]. Microneme protein 3 (MIC3) is one of the major adhesive proteins that can bind to both host and parasite cells [23]. Therefore, it is used as an antibody to detect *T. gondii*. The hematology and serum biochemical profile can be used to quickly and accurately assess the status of an animal’s health [24]. Furthermore, the biochemical profile can support the molecular understanding of the host-parasite relationship and accurate descriptions of disease [25]. These values are also critical in determining an animal’s natural physiological state, nutritional status, and pathological condition [26,27]. In a recent study, Mahmood [28] looked at the effect of *T. gondii* on hematological, biochemical, and immunological parameters in pregnant women. Infected women had higher white blood cell (WBC) counts, alanine aminotransferase (ALT), aspartate aminotransferase (AST), ALP activities, urea and creatinine concentrations, and interleukin (IL)-6 and IL-10 levels, and lower hemoglobin (HB) and packed cell volume levels.

To the best of our knowledge, no research on the impact of toxoplasmosis on the hematology and serum biochemistry of camels in Mianwali district has been conducted to date. Therefore, this study was planned with the objectives of testing seroprevalence, hematology, and serum biochemistry in the camel population in Mianwali district. The disease-related risk factors in the study area were also observed.

## Materials and Methods

### Ethical approval and Informed consent

Ethical approval for the current study was obtained from the Divisional In-charge of Disease Investigation & Control Office of Livestock & Dairy Development Department, Sargodha Division, Punjab, Pakistan. Before the sampling, verbal permission was taken from the camel owners after being briefed on the objective of the study and the blood collection technique. Furthermore, all necessary information about the farmers and their camels were carefully documented.

### Study period and location

**Figure-1:**
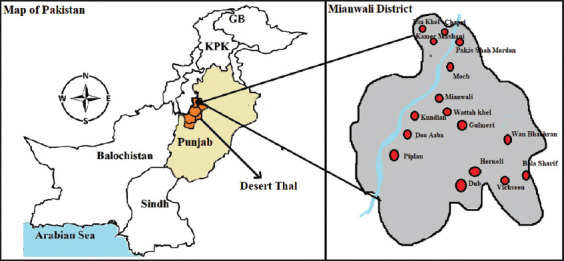
Map shows the Mianwali district’s sampling areas located northwest of desert Thal in Punjab, Pakistan. [Source: Humdata.org].

The district of Mianwali is situated in the province of Punjab, Pakistan, in the northwestern corner, with latitude 32.585411 and longitude 71.54361700000004. Attock district is in the north, Laki Marwat and Karak districts are in the northwest, and Bhakkar district is in the south. Chakwal and Khushab districts are in the east, while D.I. Khan is in the west. The Indus River runs through the district, starting in the north and splitting it into two unequal parts (Figure-1). The average high temperature per year is recorded as 47°C, while the average low temperature per year is 19°C. The mean yearly rainfall is 3.3 mm and maximum rainfall occurs in July, that is, 6.6 cm. Vegetation type of Mianwali includes wheat, barley, oat, mustard, Eruca, fennel, peanut, mung, and mash. Due to ruthless cutting of forest for fuel and timber purposes, the forests covered area is very low. Mostly the area is semi-arid, very small area is irrigated and source of the irrigation is the canals of river Indus. Mianwali has 905,142 animals, of which 867 are camels. From April 2017 to March 2018, a convenient type of sampling of camels in the district Mianwali was conducted (Table-1).

**Table-1:** Overall prevalence of *T.* *gondii* in camels of district Mianwali, Pakistan (n=350).

Characteristics	Frequency (%)	Toxoplasmosis		p-value

		Positive (%)	Negative (%)	
Gender				
Male	223 (63.7)	112 (50.2)	111 (49.8)	<0.001
Female	127 (36.3)	21 (16.5)	106 (83.5)	
Age				
≤3 years	91 (26.0)	21 (23.1)	70 (76.9)	<0.001
4-6 years	169 (48.3)	55 (32.5)	114 (67.5)	
≥7	90 (25.5)	57 (63.3)	33 (36.7)	
Breed				
Barela	268 (76.6)	109 (40.7)	159 (59.3)	0.063
Marecha	82 (23.4)	24 (29.3)	58 (70.73)	
Reproductive status of female camels				
Pregnant	25 (19.68)	7 (28)	18 (72)	0.285
Non-pregnant	92 (72.44)	8 (8.70)	84 (91.30)	
Aborted	10 (7.90)	6 (60)	4 (40)	<0.001
Non-aborted	117 (92.10)	15 (12.82)	102 (87.18)	
Purpose				
Drought/meat	324 (92.6)	125 (38.58)	199 (61.42)	0.430
Milk production	26 (7.4)	8 (30.77)	18 (69.23)	
Camel production system				
Nomadic	24 (6.9)	18 (75)	6 (25)	<0.001
Non-nomadic	326 (93.1)	115 (35.28)	211 (64.72)	

*T. gondii=Toxoplasma gondii*

### Questionnaire-based surveillance

For data collection, a questionnaire was created with open-ended and closed-ended questions and all possible determinants associated with the host, agent, and atmosphere. Formal and informal testing approaches were used for questionnaire development. In total, 350 camels (127 females and 223 males) were included in the study. The animals were divided into three age groups: (1) ≤3 years old; (2) 4–6 years old; and (3) ≥7 years old. The reproductive status of female camels was also registered so that non-pregnant, pregnant, and abortion affected camels could be compared. The influence of breed, production systems (nomadic/non-nomadic), and the purpose of producing camels also were investigated (milk, meat, and draught).

### Blood collection and sera isolation

Each camel was properly restrained and 5 mL of blood was collected from the jugular vein through a 10 mL sterile syringe. The collected sample was directly transferred to the vacutainers without additives (Improvacuter, China). The pure yellow-colored serum was obtained after centrifugation and used for further processing.

### Preparation of MIC3 protein

The previously described method of Jiang *et al*. [29] was used to purify recombinant MIC3 protein. After induction for 4 h with isopropyl-D-thiogalactopyranoside, the bacteria (*E. coli*) that expressed MIC3 protein were harvested. The cells were resuspended in phosphate-buffered saline (pH 7.4) containing 0.5% Triton X-100, 0.1% lysozyme, and 2% deoxycholic acid sodium, and then ultrasonically lysed in an ice bath.

### Indirect ELISA

The method described by Fatima *et al*. [30] was used to conduct indirect ELISA.

### Hematological and biochemical analyses

The Mythic Vet-18 unit was used to perform a complete blood count. The serum chemistry analysis was performed using a semi-automated chemistry analyzer (Photometer 5010v+, Robert Riele GmbH & Co KG Berlin, Germany. For a total of 20 camels (male = 10 and female = 10) positive for toxoplasmosis, hematological analyses were performed using an automated hematology analyzer Mythic 18 Vet Woodley Laboratory Diagnostics UK). For the negative control, 20 healthy camels (10 males and 10 females) were included in the study. The findings were then compared to Schalm’s Veterinary Hematology reference values for hematological parameters [31].

### Statistical analysis

IBM, SPSS V. 25.0 (IBM Corp., NY, USA) was used to analyze the results. Descriptive statistics were used to interpret the demographic data. A cross-tabulation test was used to explore the demographics and toxoplasmosis outcomes. Pearson’s Chi-squared test was performed to determine the difference among the demographic characteristics of the animals. Logistic regression analysis was performed to investigate the predictors of toxoplasmosis. Further, the significance of the difference between the stereochemistry means of normal and infected camels was determined with Student’s t-test. A 5% threshold value was set for significance for all these tests.

## Results

Of the 350 camels screened for *T. gondii*, 133 (38.0%) camels were positive (Table-1). In camels that were ≥7 years old, the prevalence rate was 63.33% (57/90), compared with younger animals (4-6 years old and ≤3 years old), which had prevalence rates of 32.54% (21/169) and 23.07% (21/91), respectively. The results also showed that animals of 4-6 years old and ≥7 years old had a high risk of toxoplasmosis (odds ratio [OR]=1.896, OR=0.991-3.630, p=0.053 and OR=5.178, CI=2.530-10.598, p*≤*0.001), respectively. The prevalence was higher in male camels (50.22%; 112/223) than in female camels (16.53%; 21/127) (p<0.001). Logistic regression analysis predicts that the risk of toxoplasmosis was 6.867 times higher in males than in females (OR=6.867, CI=3.098-15.221, p≤0.001). *T. gondii* was present in 60% of aborted female camels (n=10). The result also showed a significantly higher risk of toxoplasmosis in aborted animals compared with the control group (OR=7.348, CI=4.117-13.115, p*≤*0.001). Our findings revealed that the infection rate was higher in pregnant females (28%) than in non-pregnant females (8.70%). Furthermore, there was no significant association between the seroprevalence of *T. gondii* and reproductive status of female camels (pregnant or non-pregnant), camel breeds, and purpose of production (Table-1) (p*≥*0.05). *T. gondii* seropositivity rate was higher in the camel breed Barela (40.67%) than Marecha (29.26%) (Table-1). The statistical analyses revealed that the seroprevalence rate was significantly higher in male camels (50.2%) than females (112/223) (p≤0.001). Moreover, there was a significant association between *T. gondii* infection and camel production system; the seroprevalence rate was higher in nomadic camels (75%; 18/24) than in non-nomadic camels (p*≤*0.001). We also found that non-nomadic camels had a 5.679-fold higher risk of toxoplasmosis compared with nomadic animals (OR=5.967, CI=2.050-17.370, p≤0.001) (Table-2).

**Table-2:** Logistic regression analysis of determinants of *T. gondii* in Pakistan (n=350).

Characteristics	Negative	Positive	Odds ratio	CI (95%)	p-value
Gender					
Female	106 (30.3)	21 (6)	1	-	-
Male	111 (31.7)	112 (32)	6.867	3.098-15.221	<0.001
Age					
≤3 years	70 (76.9)	21 (23.1)	1	-	-
4-6 years	114 (67.5)	55 (32.5)	1.896	0.991-3.630	0.053
≥7 years	33 (36.7)	57 (63.3)	5.178	2.530-10.598	<0.001
Breed of camels					
Marecha	58 (70.73)	24 (29.3)	1	-	-
Barela	159 (59.3)	109 (40.7)	1.708	0.925-3.132	0.087
Reproductive status of female camels					
Non-pregnant	84 (91.30)	8 (8.70)	1	-	-
Pregnant	18 (72)	7 (28)	0.000	0.000	1.000
Non-aborted	102 (87.18)	15 (12.82)	1	-	-
Aborted	4 (40)	6 (60)	7.348	4.117-13.115	<0.001
Purpose					
Milk production	199 (61.42)	125 (38.58)	1	-	-
Drought/meat	18 69.23)	8 (30.77)	0.000	0.000	1.000
Camel production system					
Nomadic	6 (25)	18 (75)	1	-	-
Non-nomadic	211 (64.72)	115 (35.28)	5.967	2.050-17.370	0.001

*T. gondii=Toxoplasma gondii,* CI=Confidence interval

In *T. gondii*-infected camels, the lymphocyte percentage (LYMP%) 59±16.64, monocyte percentage (MONO%) 6.4±2.46, corpuscular volume (MCV µm^3^) 43.2±17.73, procalcitonin (PCT%) 0.15±0.174, mean capsular hemoglobin (MCH pg) 20.3±11.58, glucocorticoid-remediable aldosteronism (GRA×10^3^/µL) 5.45±9.92, were significantly higher (p=0.005) than in non-infected camels, and Hemoglobin (HB g/dL) 8.56±3.24 and hematocrit (HCT%) 21.8±10.83 values were significantly (p=0.005) lower; non-significant differences were observed for the WBC count (×10^3^/µL) 13.03±13.17, platelets (PLT×10^3^/µL) 232.9±260.32, and red blood cells (RBCs;×10^6^/µL) 19.37±116.34 observed in in comparison to the non-infected camels. Furthermore, significant effects on the values of MON (×10^3^/µL) 0.9±1.92, granulocyte percentage (GRA%) 34.5±17.13, mean capsular hemoglobin concentration (MCHC g/dL) 46.4±19.02, mean platelet volume (MPV µm^3^) 5.8±1.34, RBC distribution width (RDW%) 17.5±9.58, and platelet distribution width (PDW%) 27.9±22.33 were seen in infected camels; however, these values were within the standard range of hematological parameters for camels and were, therefore, considered as normal (Table-3).

**Table-3:** The mean values of hematological parameters in infected *T. gondii* camels.

Parameters of hematology	Control/normal range values	Normal/non-infected camel values Mean±SD	*T. gondii*-infected camel’s values Mean±SD	p-value
WBCs (×10^3^/µL)	7-15	11.00±5.66	13.03±13.17	0.075
LYMP (×10^3^/µL)	3-7	5.00±2.83	7.1±6.99	<0.001
MON (×10^3^/µL)	0.5-3	1.75±1.77	0.9±1.92	<0.001
GRA (×10^3^/µL)	1-4	2.50±2.12	5.45±9.92	0.001
LYMP%	25-50	37.50±17.68	59±16.64	<0.001
MONO%	2-6	4.00±2.83	6.4±2.46	<0.001
GRA%	12-40	26.00±19.80	34.5±17.13	<0.001
RBCs (×10^6^/µL)	7.5-12	9.75±3.18	19.37±116.34	0.339
HB (g/dL)	12-17	14.50±3.55	8.56±3.24	<0.001
HCT (%)	25-36	30.50±7.78	21.8±10.83	<0.001
MCV (µm^3^)	32-40	36.00±5.66	43.2±17.73	<0.001
MCH (pg)	12.5-16.5	14.50±2.83	20.3±11.58	<0.001
MCHC (g/dL)	42-50	46.00±5.66	46.4±19.02	<0.001
RDW (%)	16-20	18.00±2.83	17.5±9.58	<0.001
PLT (×10^3^/µL)	150-400	275.00±176.78	232.9±260.32	0.062
MPV (µm^3^)	3.5-6.5	5.00±2.12	5.8±1.34	<0.001
PCT (%)	0.02-0.018	0.019±0.00141	0.15±0.174	<0.001
PDW (%)	35-65	50.00±21.21	27.9±22.33	<0.001

*T. gondii=Toxoplasma gondii,* GRA=Glucocorticoid-remediable aldosteronism, LYMP=Lymphocyte percentage, MONO%=Monocyte percentage, WBC=White blood cell, RBC=Red blood cell, MCHC=Mean capsular hemoglobin concentration, MPV=Mean platelet volume, RDW=RBC distribution width, PDW=Platelet distribution width, HB=Hemoglobin

**Figure-2:**
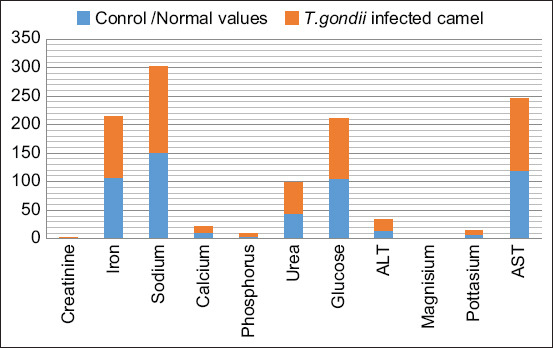
Difference in serum biochemistry values *Toxoplasma gondii*-infected camels and healthy camels.

In *T. gondii*-infected camels, liver enzyme parameters, including serum values ALT (U/L) 19.26±1.49 and AST (U/L) 125.5±2.75 as well as urea (mg/dL) 53.9±4.50 and potassium (mg/dL) 7.30±0.81 levels, were significantly (p=0.005) increased, whereas values of magnesium (mg/dL) 2.5±0.56 and glucose (mg/dL) 105.4±18.44 were found to be significantly decreased and there was a non-significant effect on the values of sodium (mmol/dL) 151.4±11.24 and iron (µg/dL) 107.5±39.31. Although statistically significant effects were noted on creatinine (mg/dL) 0.72±0.351, phosphorus (mg/dL) 4.3±0.75, and calcium (mg/dL) 10.3±1.29, these values were within the standard ranges for camels and were, therefore, considered normal (Table-4 and Figure-2).

**Table-4:** The mean values of determinant parameters for serum biochemistry of *T. gondii*-infected camels.

Parameters of serum chemistry	Control/normal range values	Normal/non-infected camel values Mean±SD	*T. gondii-*infected camels Mean±SD	p-value
Creatinine (mg/dL)	0.7-1.4	1.05±0.49	0.72±0.351	<0.001
Iron (µg/dL)	82-135	104.50±31.82	107.5±39.31	0.384
Sodium (mmol/dL)	145-155	150.00±7.07	151.4±11.24	0.146
Calcium (mg/dL)	8-10.3	9.15±1.63	10.3±1.29	<0.001
Phosphorus (mg/dL)	3.2-5.9	4.55±1.91	4.3±0.75	<0.001
Urea (mg/dL)	15-45	30.00±21.21	53.9±4.50	<0.001
Glucose (mg/dL)	106-119	112.50±9.19	105.4±18.44	<0.001
ALT (U/L)	11-14.5	12.75±2.47	19.26±1.49	<0.001
Magnesium (mg/dL)	1.82-3.77	2.80±1.38	2.5±0.56	<0.001
Potassium (mg/dL)	4.6-7.1	5.85±1.77	7.3±0.81	<0.001
AST (U/L)	60-120	90±42.42	125.5±2.75	<0.001

*T. gondii=Toxoplasma gondii*, ALT=Alanine aminotransferase, AST=Aspartate aminotransferase

## Discussion

The aim of the present study was to estimate the seroprevalence of *T. gondii* on camels and its effects on the hematology and biochemistry parameters of infected animals in Mianwali district and the risk factors associated with *T. gondii* infection in the studied population. The overall seroprevalence of *T. gondii* was found to be 38%. The prevalence was higher in male camels (50.2%) than in female camels (16.5%). Our findings are in line with a previous study (40.1%) recorded by Fatima *et al*. [30]. Furthermore, the seroprevalence in our study was slightly higher than that found in two different studies of Saudi Arabia (35.8% and 34.2%), Somalia (34.4%), and Africa (36%) [32-35]. However, our current findings are significantly lower than those published from the Czech Republic (69%) [36], Iran (65%) [37], and Turkey (91%) [17]. The current variation in the seroprevalence of toxoplasmosis may be due to region, climate effects, management system, age, and analytical techniques used in the study [30,38-41].

Our findings for male camels were comparable with the results of studies conducted in the Taif (56.7%) and Jizan (54.2%) areas of Saudi Arabia [33]. The current difference in prevalence rates could be attributed to the fact that most farmers use males as draught animals for goods transportation and plowing agricultural fields. These movements of these camels into field areas and their browsing habits increase the risk of *T. gondii* infection through the inhalation or ingestion of sporulated oocysts spread by cats in the fields [13,42]. The current study found that the seroprevalence rate of *T. gondii* was directly related to the age of camels. Furthermore, as camels aged, there was a significant increase in seroprevalence rate. The highest prevalence rate was found in camels of ≥7 years old. These findings were consistent with those reported by Fatima *et al*. [30] which indicated that the seroprevalence of *T. gondii* infection was higher in older camels (70.6%) than that of youngers (4–6 years; 33.1%, ≤ 3 years; 18.5%). The current high seroprevalence rate of *T. gondii* in older camels is due to the camels’ movement to agriculture fields and feeding in the field areas, where they are more exposed to *T. gondii* compared with younger camels [43]. Moreover, our findings substantiated the research conducted in Algeria, Egypt, Saudi Arabia, and Pakistan, which found that the prevalence rate of *T. gondii* increased significantly with age [44-47]. The prevalence rate was higher in Barela camels (40.67%) than in Marecha camels. As *T. gondii* seroprevalence rate is known to vary between different animal breeds [48], this may be one factor responsible for the variation in the findings of the current study. Moreover, the Barela is a potential milking camel breed, and milking camels are more susceptible to *T. gondii* infection than non-milking camels (e.g., Marecha) [30,49].

*T. gondii* infection was higher in aborted female camels (60%) than in non-aborted female camels (12.82%). Furthermore, there was a statistically significant (<0.001) correlation between *T. gondii* infection and abortion. These study findings reveal that the leading cause of abortion in female camels is *T. gondii* infection [50]. The prevalence of *T. gondii* in camels used for draught and raised non-nomadically was higher, consistent with a previous study in Pakistan [30]. In our opinion, the higher prevalence rate in draught camels resulted from their exposure to *T. gondii* in fields. In contrast, the higher prevalence in non-nomadic camels was due to domestic cats contaminating their water and feed sources [43,51,52].

Hematology and biochemistry parameters are the most important physiological tools that reveal the basic information on the diagnosis and prognosis of any disease [47,53]. The hematology parameters of GRA, LYMP%, MONO%, MCV, MCH, and PCT were significantly increased (p≤0.005) in infected camels compared with non-infected camels. Thus, the findings showed that *T. gondii* infection significantly affected the normal hematology parameters in camels; LYMP%, MCH, and MCV were notably increased in infected camels [47]. Our findings also support the work of Raisinghani and Lodha [54], Partani *et al*. [55], Chaudhary *et al*. [56], Ahmad *et al*. [57], and Sazmand *et al*. [58]. Toxoplasmosis induces leukopenia [57], but the WBC% in our study was different from other studies, which may be due to sample handling procedures [59]. Similarly, the HCT and HB values of infected camels in our study were significantly decreased, which are in line with the study of Lashari *et al*. [47].

The hematological analysis revealed a significant (p≤0.05) reduction in the total RBC count and HB concentration in the infected camels. The low RBC count and HB concentration are collectively responsible for the cause of anemia in cases of toxoplasmosis infection [60]. HB and HCT were significantly (p*≤*0.05) lower than the control values. The lower values of HB indicate anemia in infected camels and low HCT values indicate a lower number of blood cells in camels. *T. gondii* infection causes anemia, which is marked by a reduction in HCT [61]. The serum biochemical analysis of infected camels reveals a significant (p≤0.05) rise in the levels of liver enzymes, such as ALT and AST. Toxoplasmosis is considered as a liver-damaging disease that causes changes in the liver metabolic processes [62-64]. The variations in the values of ALT and AST are an excellent indicator of hepatic damage. Usually, these enzymes are present in liver, where they are involved in the metabolic processes of amino acids for energy production. However, in †he case of hepatocellular injury, these enzymes may leak into the bloodstream, resulting in their increased activity [65]. The results of the current study show an increase in ALT values that were similar to that previously reported in *T. gondii*-infected camels in Pakistan [47]. The increase in the AST level is attributed to muscular and liver damage. Our findings contradict the AST results of Lashari *et al*. [47], but completely agreed with the findings of Muhsin *et al*. [66] and El-Sayed *et al*. [67]. Moreover, in the current study, higher values of potassium were observed, which increased the risk of renal dysfunction involving creatinine and blood urea nitrogen [61]. Our finding of high potassium level was similar to the results reported in toxoplasmosis-infected cats [61]. In the current study, the glucose values were significantly lower, indicating that *T. gondii* uses excessive glucose for metabolism; these findings agreed with the results of Lashari *et al*. [47].

Some other studies also supported our findings, with increased values of ALT and AST observed in *T. gondii* infection in other species, including gerbils, goats, dogs, and humans (a study in women only) [68-72]. Increased ALT and AST levels indicate liver dysfunction, which is the primary cause of enzymatic overproduction in the bloodstream [73]. In the current study, the increased urea level was in line with the findings of Lashari *et al*. [47]. The increase in potassium level was similar to the study by Iewida *et al*. [61] in *T. gondii*-infected cats. In contrast, a significant decrease in glucose level was observed in infected camels, which agreed with the study of Lashari *et al*. [47] and supported the study of Anosa [74], in which the researcher claimed that the parasite consumes glucose during metabolic processes.

## Conclusion

The current study confirmed the significant effects of *T. gondii* infection on hematological and serum chemistry parameters in camels. Further, a direct relationship between camel age and *T. gondii* infection rate was found. The emergence of a high seroprevalence rate of *T. gondii* in camels is a serious public health concern. Therefore, a collaborative effort between public health bodies and veterinary authorities is required to conduct epidemiological studies in various species rearing areas, from which potential eradication and control strategies against *T. gondii* spread can be introduced. Although the current study yielded some interesting results, it has limitations, including focusing on only one district and small sample size to analyze the prevalence rate and its association with breed, gender, age, reproductive status, and camel production system.

## Authors’ Contributions

AM, TF, AS, and FMK: Conceptualization, design, sample collection, and data analysis. SF, AS, and SB: Performed the data entry and statistical analysis. AS, ZUA, and SR: Drafted the manuscript. MHE, LTS, IK, and WT: Revised and finalised the manuscript. All authors read and approved the final manuscript.
